# Preparation of uniform magnetic recoverable catalyst microspheres with hierarchically mesoporous structure by using porous polymer microsphere template

**DOI:** 10.1186/1556-276X-9-163

**Published:** 2014-04-04

**Authors:** Lianbing Ren, Chao Teng, Lili Zhu, Jie He, You Wang, Xinbing Zuo, Mei Hong, Yong Wang, Biwang Jiang, Jing Zhao

**Affiliations:** 1 Shenzhen Key Lab of Nano-Micro Materials Research, School of Chemical Biology & Biotechnology, Peking University Shenzhen Graduate School, Shenzhen 518055, China; 2 Shenzhen Middle School, Shenzhen 518000, China; 3 Shenzhen State High-tech Industrial Innovation Center, Shenzhen 518000, China; 4 State Key Laboratory of Pharmaceutical Biotechnology, School of Life Sciences, Nanjing University, Nanjing 210093, China

**Keywords:** Magnetic nanoparticles, Gold nanoparticles, Mesoporous silica microspheres, Hierarchical pores, Catalysts, 61.46.-w, 75.75.-c, 81.07.-b

## Abstract

Merging nanoparticles with different functions into a single microsphere can exhibit profound impact on various applications. However, retaining the unique properties of each component after integration has proven to be a significant challenge. Our previous research demonstrated a facile method to incorporate magnetic nanoparticles into porous silica microspheres. Here, we report the fabrication of porous silica microspheres embedded with magnetic and gold nanoparticles as magnetic recoverable catalysts. The as-prepared multifunctional composite microspheres exhibit excellent magnetic and catalytic properties and a well-defined structure such as uniform size, high surface area, and large pore volume. As a result, the very little composite microspheres show high performance in catalytic reduction of 4-nitrophenol, special convenient magnetic separability, long life, and good reusability. The unique nanostructure makes the microspheres a novel stable and highly efficient catalyst system for various catalytic industry processes.

## Background

Owing to their higher catalytic activity, better selectivity, and longer stability than Pd and Pt catalysts, the catalysis of gold nanoparticles (Au NPs) in liquid-phase reactions has become the subject of increasing interest in recent years [1-15]. It has been proven that smaller Au NPs show higher catalytic activity as they have much greater surface to volume ratio [16-18]. However, small Au NPs easily aggregate to minimize their surface area, resulting in a remarkable reduction in their catalytic activity [19,20]. Immobilizing Au NPs onto solid supports to form composite catalysts is regarded as a practical strategy to solve this problem [21-26]. For liquid-phase reactions, the catalysts need to be separated easily from the mixture for recycling. Among various kinds of supports with different nanostructures, porous magnetic composite nanomaterials have aroused considerable attention since they could satisfy two requirements simultaneously: high surface area and facile recycle [22-24,27-31]. The high surface area comes from the hierarchically porous structure which provides enough exposure of the composite catalysts to the reactants. The facile recyclability results from the magnetic nature of the composite catalysts, which enables fast separation of the solid catalysts from the reaction mixture by applying an external magnet.

Several strategies have been developed to immobilize Au NPs onto/into the magnetic composite supports [27-35]. Generally, Au NPs are pre-synthesized and then incorporated into the modified supports. Ge et al. reported the synthesis of a nanostructured hierarchical composite composed of a central magnetite/silica composite core and many small satellite silica spheres [6]. Au NPs were immobilized on the silica satellites through gold-amine complexation. The obtained supported gold catalysts showed fast magnetic separation ability and high catalytic activity for 4-nitrophenol reduction. Deng et al. deposited Au NPs onto modified Fe_3_O_4_@SiO_2_ microspheres followed by a surfactant-assembly sol-gel process and synthesized multifunctional Fe_3_O_4_@SiO_2_-Au@mSiO_2_ microspheres with well-defined core-shell nanostructures, confined catalytic Au NPs, and accessible ordered mesopore channels [7]. However, most of these methods are tedious and time-consuming. Recently, Zheng et al. successfully developed an approach to *in situ* load Au NPs on Fe_3_O_4_@SiO_2_ magnetic spheres [8]. After the Fe_3_O_4_@SiO_2_ magnetic nanoparticles were firstly prepared, AuCl_4_^-^ was introduced to the surface and then reduced by Sn^2+^ species that were linked to the surface of the Fe_3_O_4_@SiO_2_ precursor. The synthesis step and the reaction cost were remarkably decreased. Despite of these researches, *in situ* fabrication is limited [25,36-39], and it is still a challenge to develop an efficient and facile method to immobilize Au NPs in solid magnetic supports without compromising the catalytic activity.

**Figure 1:**
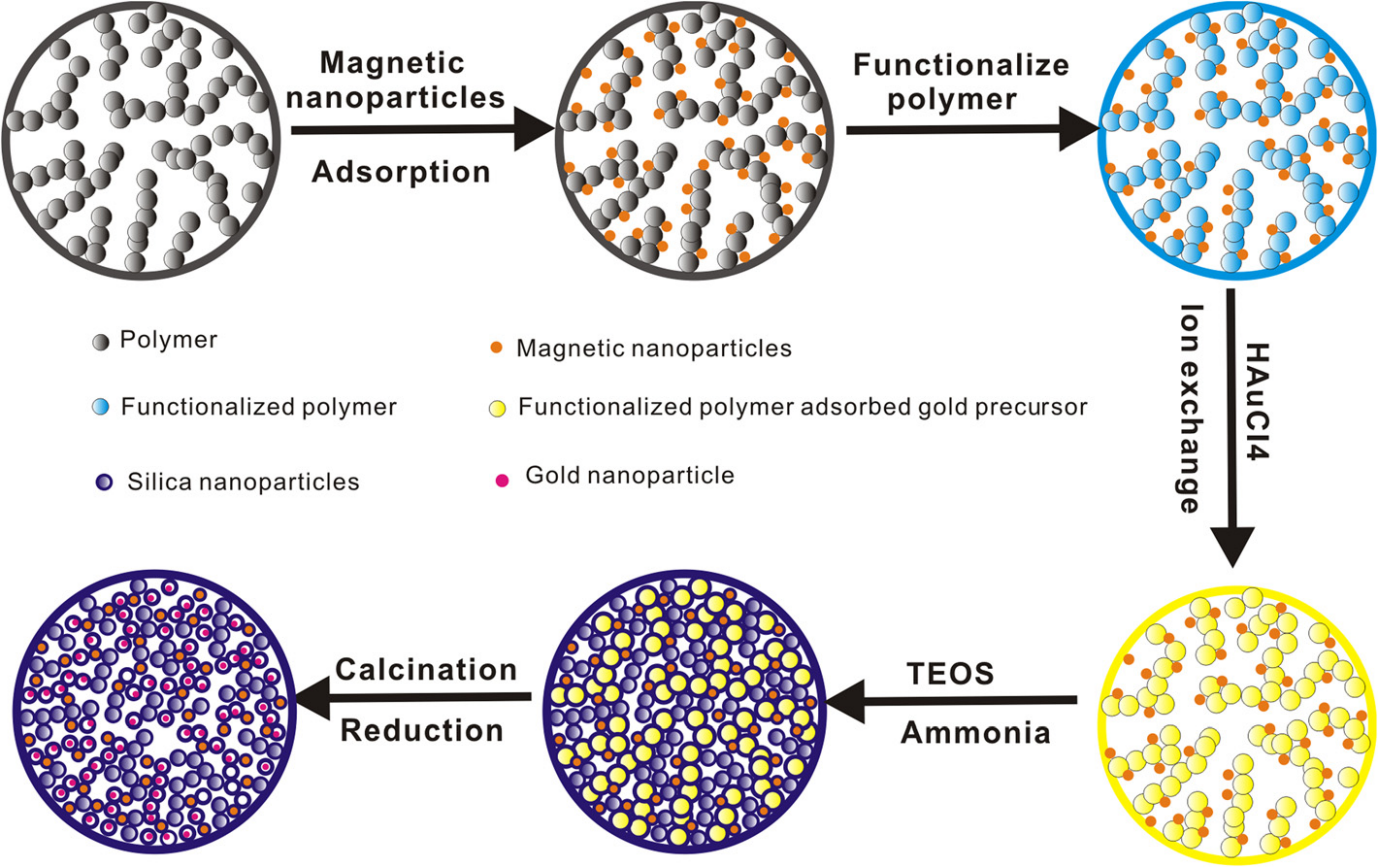
Schematic illustration of the synthetic procedure of porous silica microspheres embedded with magnetic and gold nanoparticles.

To overcome the above problems, herein, on the basis our group's previous work of incorporating magnetic nanoparticles into porous silica microspheres, we report an efficient method for the synthesis of uniform multifunctional mesoporous silica microspheres incorporated with magnetic and gold nanoparticles by using porous polymer microspheres as template. The preparation strategy is shown in Figure 1. Firstly, porous glycidyl methacrylate (GMA) cross-linked with ethylene glycol dimethacrylate (EGDMA) polymer P(GMA/EGDMA) microspheres doped with magnetic nanoparticles (γ-Fe_2_O_3_) are synthesized via the method in our previous report. Secondly, the surface of the porous magnetic polymer microspheres are modified by a quaternary amine via ring-opening reaction of epoxide groups of GMA with trimethylamine (TMA). Thirdly, the gold precursor (AuCl_4_^-^) is adsorbed onto TMA-treated magnetic polymer composite microspheres through the ion exchange between quaternary ammonium ions and AuCl_4_^-^. Then, the silica nanoparticles are deposited into the channel of magnetic P(GMA/EGDMA)-N^+^/AuCl_4_^-^ composite microspheres through sol-gel reaction with the silica precursor tetraethylorthosilane (TEOS). Finally, uniform mesoporous silica microspheres embedded with magnetic and gold nanoparticles, designated as γ-Fe_2_O_3_/Au/mSiO_2_, are obtained after calcinations to remove the polymer template and organic agents. The designed multifunctional microspheres possess uniform particle size, large magnetization, hierarchical mesopores, and stably confined but exposed active metal nanoparticles. The multifunctional porous microspheres show excellent catalytic performance towards the reduction of 4-nitrophenol by excess sodium borohydride (NaBH_4_) in aqueous solution and could be very useful in various catalytic reductions. With an external magnetic field, the catalyst can be easily recycled. Long lifetime and high reusability are demonstrated with negligible decrease in the catalytic performance after use for more than ten times.

## Methods

### Materials

The silica precursor tetraethylorthosilane (TEOS) was purchased from Alfa Aesar (Beijing, China). The template polymer microspheres are a polymer of glycidyl methacrylate (GMA) cross-linked with ethylene glycol dimethacrylate (EGDMA) supplied by Nano-Micro Technology Company (Jiangsu, China). Ferric chloride hexahydrate (FeCl_3_ · 6H_2_O), sodium oleate, trimethylamine (TMA) hydrochloride, sodium hydroxide, ammonium hydroxide (28% aqueous solution), and ethanol were purchased from Shanghai Chemical Reagent Corp. (Shanghai, China). Hexanes, chloroform, sodium borohydride (NaBH_4_), 4-nitrophenol (4-NP), and 1-octadecene were purchased from Alfa Aesar. Anhydrous alcohol and chloroauric acid tetrahydrate (HAuCl_4_ · 4H_2_O) were purchased from Sinopharm Chemical Reagent Co., Ltd. (Shanghai, China) Water was purified by distillation followed by deionization using ion exchange resins. Other chemicals were of analytical grade and used without any further purification.

### Synthesis of magnetic γ-Fe_2_O_3_ nanoparticles

Monodisperse magnetic γ-Fe_2_O_3_ nanoparticles were synthesized through the thermal decomposition of organometallic precursors with modifications [19]. Typically, 10 g of ferric chloride hexahydrate and 35 g of sodium oleate were dissolved in a mixture of 90 ml of ethanol, 70 ml of water, and 130 ml of hexane. The mixed solution was heated to 70°C for 4 h. The resulting ferric oleate was washed four times with 50 ml of distilled water and dried at 50°C. Then, 36 g of the iron-oleate complex synthesized as described above and 5.7 g of oleic acid were dissolved in 200 g of 1-octadecene at room temperature. The reaction mixture was heated to 320°C with a constant heating rate of 3.3°C/min and then kept at 320°C for 30 min. When the reaction temperature reached 320°C, the initial transparent solution became turbid and brownish black. The resulting solution containing the nanoparticles was then cooled to room temperature, and 500 ml of ethanol was added to the solution to precipitate the nanoparticles, which were subsequently separated by centrifugation. The weight of dry oleate-capped magnetic nanoparticles was 8.2 g.

### Preparation of magnetic polymer composite microspheres doped with γ-Fe_2_O_3_ nanoparticles

Magnetic nanoparticles (0.2 g) were added into 50 ml of toluene. After ultrasonic treatment in water bath for 1 h, a homogeneous yellow solution was obtained. Another 100 ml toluene containing 2 g of P(GMA-EGDMA) microspheres was prepared. Under stirring, the magnetic nanoparticle solution was added into the polymer microsphere solution. After 2 h, magnetic nanoparticle-embedded porous polymer microspheres were filtrated and washed repeatedly with toluene and ethanol. The brown magnetic polymer composite microspheres were dried at 50°C under vacuum.

### Surface modification of magnetic polymer composite spheres

Brown composite spheres (2 g) were dispersed in 250-ml mixture of ethanol and water (volume ratio = 2:1). Then, 2 g of trimethylamine hydrochloride and 1 g of sodium hydroxide were added to the mixture solution. After the resulting mixture was stirred in water bath at 50°C for 24 h, the resulting TMA-treated magnetic P(GMA-EGDMA) composite microspheres were filtrated and washed repeatedly with distilled water. The brown functionalized magnetic polymer composite microspheres were dried at 50°C under vacuum.

### Functionalized magnetic polymer composite microspheres adsorbed with gold precursors

TMA-treated magnetic P(GMA-EGDMA) composite microspheres (1.0 g) were added to a 100-ml round-bottomed flask, and then 50 ml deionized water and 5 ml aqueous HAuCl_4_ · 4H_2_O (1.0 wt%) were subsequently added at room temperature with mechanical stirring. After 4 h, the reddish brown precipitate was recovered by a magnet and washed with water for five times. The precipitate for further characterization was dried at 60°C for 6 h.

### Preparation of mesoporous silica microspheres embedded with γ-Fe_2_O_3_ and Au nanoparticles

In a 250-ml three-necked, round-bottomed flask equipped with a mechanical stirrer, 80 ml of ethanol and 20 g of water were placed. With vigorous stirring in the flask, 0.5 g of magnetic P(GMA-EGDMA)-N^+^/AuCl_4_^-^ composite microspheres and 2 ml of ammonia hydroxide were introduced over a period of 0.5 h. A 10% TEOS solution (in ethanol) of 30 ml was then added dropwise into the mixture in 1.5 h. The sol-gel transformation of TEOS to silica in the pore of the composite polymer microspheres was carried out at 30°C for 24 h. The brown γ-Fe_2_O_3_/polymer/gold/silica microspheres obtained were washed repeatedly with ethanol and distilled water before being dried at 50°C overnight. The dried microspheres were calcined at 600°C for 10 h (ramp rate of 10°C/min) under air. After calcination, yellow hierarchically porous silica microspheres embedded with γ-Fe_2_O_3_ and Au nanoparticles were obtained.

### Catalytic reduction of 4-NP

The reduction of 4-NP by NaBH_4_ was chosen as a model reaction for investigating the catalytic performance of the porous SiO_2_/Au/γ-Fe_2_O_3_ composite microspheres. Typically, aqueous solution of 4-NP (5 mM, 1 ml) was mixed with fresh aqueous solution of NaBH_4_ (0.4 M, 5 ml). Two milliliters of aqueous suspension of the SiO_2_/Au/γ-Fe_2_O_3_ composite microspheres (1.0 mg) was rapidly added. Subsequently, 2 ml aqueous suspension at a given interval was sampled and filtered through 0.45-μm membrane filters. The UV-visible absorption spectra of the filtrates were recorded at room temperature.

### Characterizations

The morphology and structure of the porous SiO_2_/Au/γ-Fe_2_O_3_ composite microspheres were studied using a field emission scanning electron microscope (FESEM; Hitachi S4800, Chiyoda-ku, Japan) and a transmission electron microscope (TEM; FEI Tecnai G2, Hillsboro, OR, USA). The particle hydrodynamic size was measured by using a Beckman Coulter Counter laser size analyzer (Multisizer 3, Fullerton, CA, USA). The thermogravimetric analysis was conducted on a DuPont TGA 2050 (Wilmington, DE, USA), with a temperature ramp of 10°C/min. The magnetization curve was measured at room temperature under a varying magnetic field with a vibrating sample magnetometer (ISOM, UPM, Madrid, Spain). N_2_ adsorption and desorption isotherms were measured at 77 K on a Micromeritics TriStar II 3020 (Norcross, GA, USA). The X-ray diffraction (XRD) pattern of the prepared powder sample was collected using a Rigaku D/Max-2200PC X-ray diffractometer with Cu target (40 kV, 40 mA, Shibuya-ku, Japan). The γ-Fe_2_O_3_ content in the silica microspheres was determined by atomic absorption spectroscopy (AAS; PerkinElmer 3110, Waltham, MA, USA) of an extract from the sample obtained with dilute HCl (1:1) and HF (1:1) at 80°C for 6 h. UV absorbance spectra were measured using a NanoDrop 2000 spectrophotometer (Thermo Fisher Scientific, Waltham, MA, USA).

## Results and discussion

### Characterization of γ-Fe_2_O_3_/Au/mSiO_2_ microspheres

**Figure 2:**
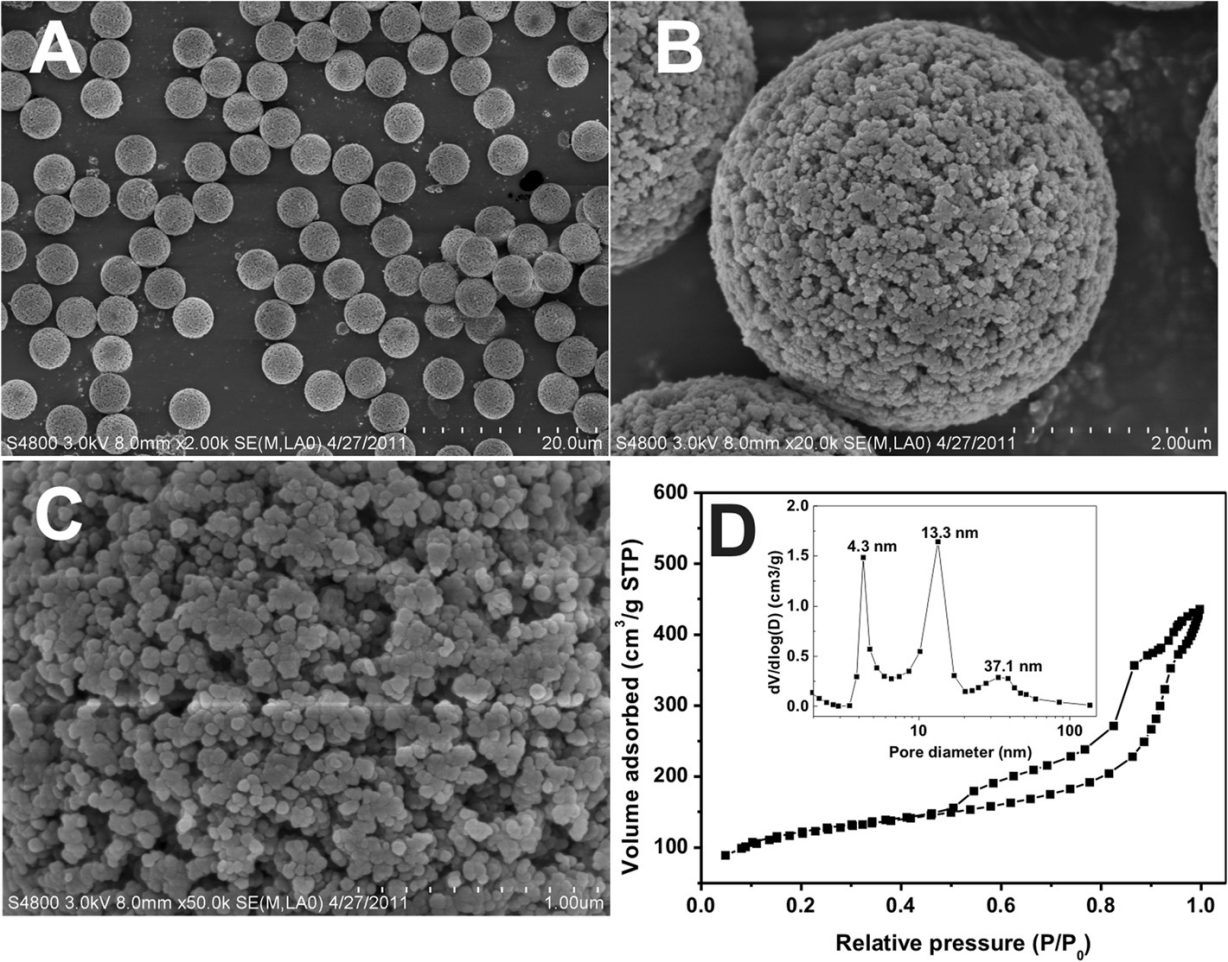
**SEM images, N**_**2 **_**adsorption/desorption isotherms, and pore size distributions of the hybrid microspheres. (A-C)** SEM images of the porous γ-Fe_2_O_3_/Au/mSiO_2_ hybrid microspheres with different magnifications. **(D)** N_2_ adsorption/desorption isotherms and pore size distributions (the inset figure) of the porous γ-Fe_2_O_3_/Au/mSiO_2_ hybrid microspheres.

Figure 2A,B,C shows representative SEM images of the synthesized porous silica microspheres incorporated with γ-Fe_2_O_3_ and gold nanoparticles. The composite microspheres are highly monodisperse with the diameter about 4.4 μm which are assembled by nanoparticles of about 30 nm. The surface morphology of the composite microspheres is a porous structure which is similar to that of the porous polymer template microspheres (Additional file 1). These similar porous microsphere morphologies indicate that the silica nanoparticles are deposited in the matrix of the polymer template in the process of sol-gel reaction of TEOS. Nitrogen adsorption measurement (Figure 2D) shows that the pore structure of composite microspheres is mesoporous. The insert pore size distribution curve shows that the primary, secondary, and tertiary pore diameters are centered at 4.3, 13.3, and 37.1 nm, respectively, indicating that the composite microspheres have hierarchical mesoporous structures on at least three levels. The BET surface area and pore volume are 363.2 m^2^/g and 0.57 cm^3^/g, respectively. The mechanism for the formation of a hierarchical mesoporous structure of the composite microsphere is similar to that of silica microspheres which has been proven in our previous report [29]. The pores at 13.3 and 4.3 nm are formed by the shrinkage of the porous polymer matrix template during calcination and the permeation of the TEOS molecules in the polymer template. The largest pore size, 37.1 nm, is at the grain boundary between silica nanoparticles.

**Figure 3:**
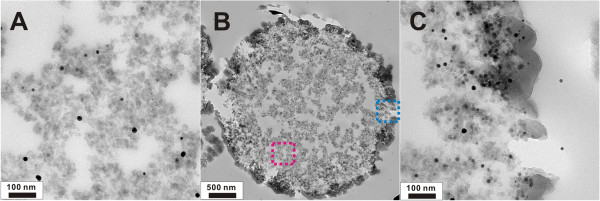
**TEM images. (A)** The central area (enlarged view of the pink square in **B)**. **(B)** The inner structure of the ultramicrotomed porous γ-Fe_2_O_3_/Au/mSiO_2_ hybrid microsphere. **(C)** The edge area (enlarged view of the blue square in **B)**.

The detailed inner structures of the composite microspheres have been characterized by TEM. As shown in Figure 3 of the ultramicrotomed microsphere sample, the morphology inside the microspheres is a porous structure with connecting channels similar to that on the surface. Furthermore, several metal nanoparticles about 10 to 20 nm with different image contrast, the black and gray dots, are found to be encapsulated in the whole range of the porous silica matrix, the edge area (Figure 3C) and the central area (Figure 3A). As reported in the literature, amines have been known to act both as stabilizer and as reducing agents for gold nanoparticles. Biffis and Minati reported that the tertiary amine groups could reduce Au(III) to Au(0) [40]. In our reaction process, the embedded nanoparticles might be γ-Fe_2_O_3_ and gold nanoparticles because AuCl_4_^-^ are reduced *in situ* to form gold nanoparticles by the reaction with quaternary ammonium ions on the polymer template while adsorbed magnetic nanoparticles remain unchanged in the process of calcination.

**Figure 4:**
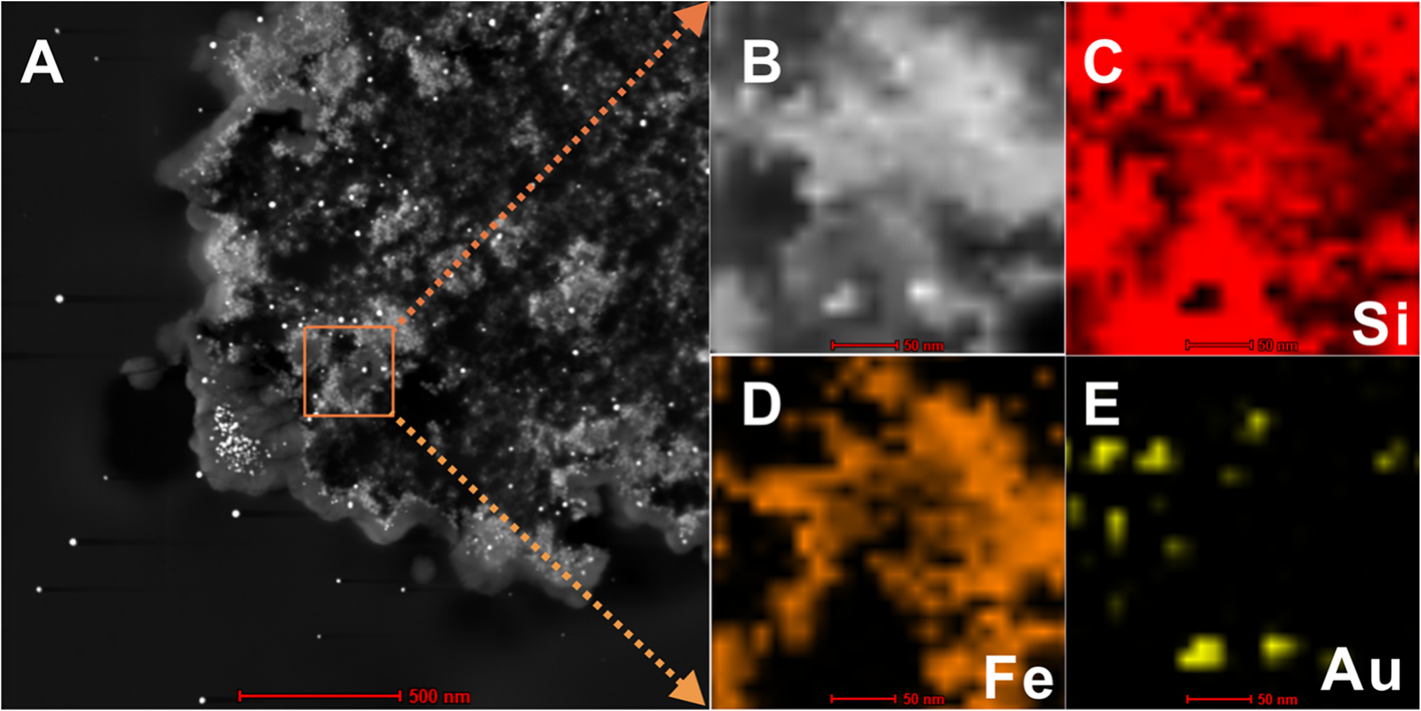
**STEM and STEM-EDX elemental mapping images. (A, B)** STEM images of the ultramicrotomed porous γ-Fe_2_O_3_/Au/mSiO_2_ microspheres. **(C-E)** STEM-EDX elemental mapping images of the selected area in Figure 4A.

**Figure 5:**
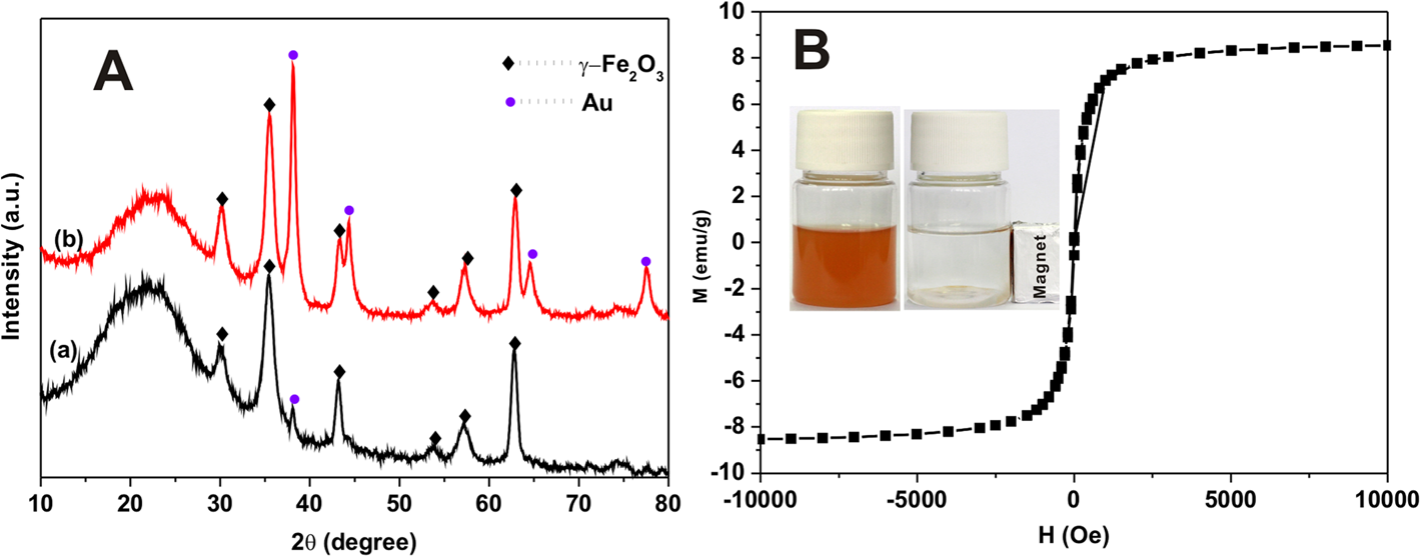
**XRD pattern and magnetic hysteresis curves of hybrid microspheres. (A)** XRD pattern of (a) γ-Fe_2_O_3_/polymer/Au/SiO_2_ and **(B)** γ-Fe_2_O_3_/Au/SiO_2_ hybrid microspheres. **(B)** Magnetic hysteresis curves of the porous γ-Fe_2_O_3_/Au/SiO_2_ hybrid microspheres. The inset is a photograph of the porous γ-Fe_2_O_3_/Au/SiO_2_ microspheres under an external magnetic field.

In order to confirm that the embedded nanoparticles are magnetic and gold nanoparticles, we use scanning transmission electron microscopy (STEM) to characterize the sample. As shown in Figure 4, nanoparticles (the bright spots) are well dispersed in porous silica microspheres. The existence of Si (SiO_2_), Fe (Fe_2_O_3_), and Au is confirmed by STEM-energy-dispersive X-ray (EDX) analysis. To further verify the formation of Fe_2_O_3_ and gold nanoparticles, Figure 5A shows the XRD patterns of the samples before and after calcination. Six characteristic diffraction peaks (2*θ* = 30.3°, 35.6°, 43.2°, 53.5°, 57.2°, and 62.9°), related to their corresponding indices ((220), (311), (400), (422), (511), and (440)), are clearly observed in Figure 5A, indicating the presence of γ-Fe_2_O_3_ in the products. The four peaks positioned at 2*θ* values of 38.2°, 44.4°, 64.5°, and 77.4° could be attributed to the reflections of the (111), (200), (220), and (311) crystalline planes of cubic Au, respectively. In addition, we find that only a weak peak (2*θ* = 38.2°) clearly shows up in Figure 5A (a), indicating that a small amount of gold precursors is reduced by quaternary ammonium ions before calcination. The process of calcination promotes the formation of gold nanoparticles. The magnetization curve of the resulting materials shows that the magnetic saturation (Ms) value is 8.4 emu/g, which indicates that γ-Fe_2_O_3_ nanoparticles are incorporated into the hybrid materials as well (Figure 5B). As shown in Figure 5B insert, the porous γ-Fe_2_O_3_/Au/SiO_2_ microspheres could be well dispersed in water to form a translucent yellowish brown solution. After applying this solution to magnetic field, the dispersed microspheres are quickly attracted to the wall of the vial close to the magnet within 1 min and the solution becomes transparent. The excellent magnetic response makes the porous γ-Fe_2_O_3_/Au/SiO_2_ microspheres easy to separate and reuse.

### Application of the porous γ-Fe_2_O_3_/Au/SiO_2_ microspheres for catalytic reduction of 4-NP

**Figure 6:**
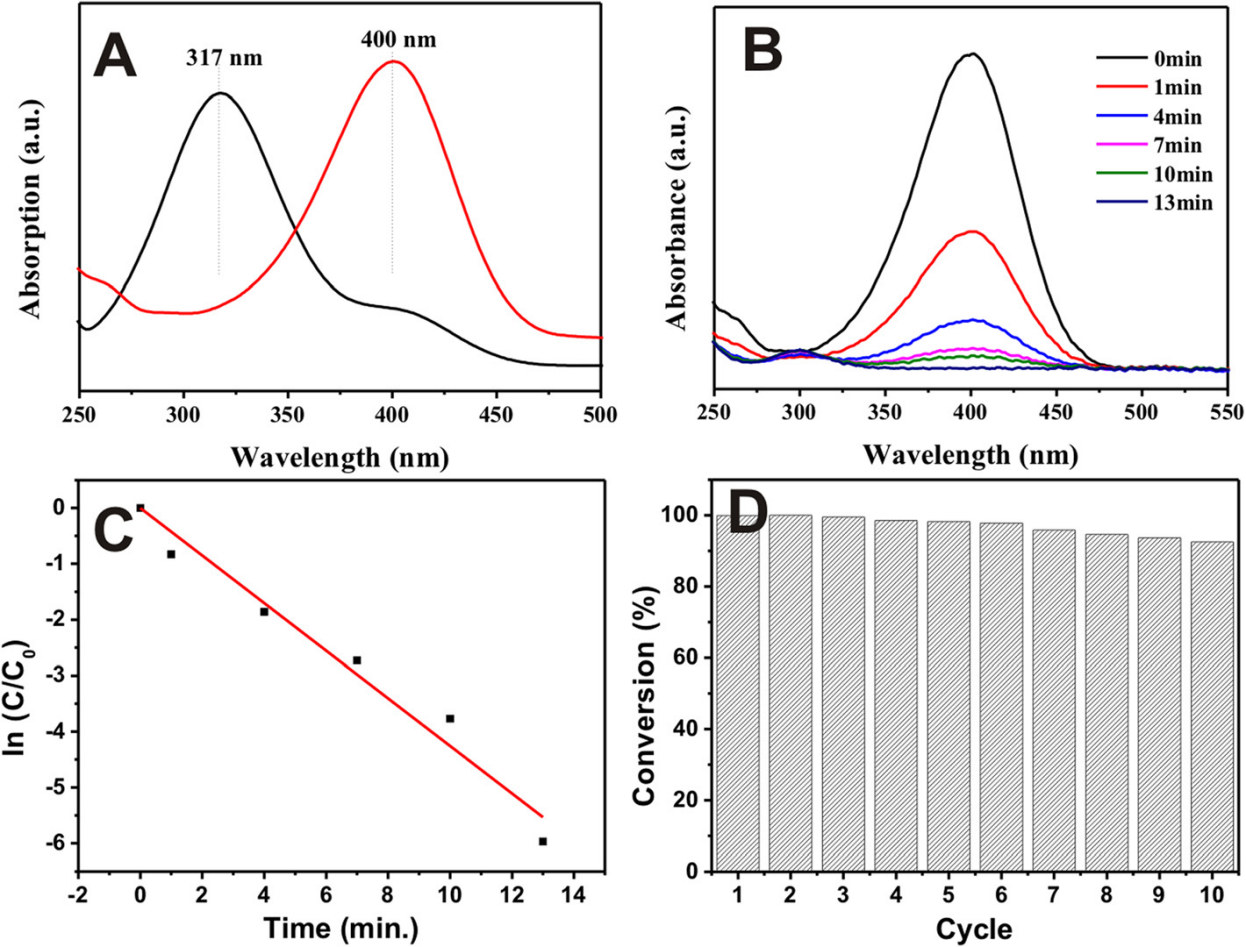
**UV-vis spectra and reduction of 4-NP, linear relationship, and reusability of the microspheres. (A)** UV-vis spectrum of 4-NP before and after adding NaBH_4_ solution. **(B)** The reduction of 4-NP in aqueous solution recorded every 3 min using the porous γ-Fe_2_O_3_/Au/SiO_2_ microspheres as a catalyst. **(C)** The relationship between ln(*C*_t_/*C*_0_) and reaction time (*t*). **(D)** The reusability of the porous γ-Fe_2_O_3_/Au/mSiO_2_ microspheres as a catalyst for the reduction of 4-NP with NaBH_4_.

4-nitrophenol (4-NP) is one of the most common organic pollutants in industrial and agricultural wastewater, while 4-aminophenol (4-AP) has important applications in many fields, such as medicine, photography, anticorrosion, etc. The conversion of 4-NP to 4-AP under the catalyst of noble metal NPs, which simultaneously realizes the degradation of 4-NP and the efficient production of 4-AP, has attracted the interest of researchers. Here, the reduction of 4-NP by NaBH_4_ is chosen as a model reaction for investigating the catalytic performance of the porous γ-Fe_2_O_3_/Au/SiO_2_ microspheres. There is no appreciable by-product formation during this reaction. The extent of the reaction could be determined by measuring the change in UV-visible (UV-vis) absorbance at 400 nm. As shown in Figure 6A, the 4-NP solution shows adsorption at approximately 317 nm. After addition of NaBH_4_, the adsorption maximum shifts to 400 nm immediately, due to the formation of 4-nitrophenolate. No change is observed after standing for a long time, indicating that the reduction does not proceed without a catalyst.

A small quantity (1.0 mg) of the γ-Fe_2_O_3_/Au/SiO_2_ microspheres is added and the adsorption peak at 400 nm significantly decreases as the reaction proceeds, revealing the reduction of 4-NP to form 4-AP. Figure 6B shows the UV-vis spectra as a function of reaction time for a typical reduction process. The full reduction of 4-NP by NaBH_4_ is completed within approximately 13 min, and the bright yellow solution gradually becomes colorless. Linear relationships between ln(*C*_t_/*C*_0_) and reaction time are obtained in the reduction catalyzed by the γ-Fe_2_O_3_/Au/mSiO_2_ microspheres (Figure 6C), which well matches the first-order reaction kinetics. The rate constant *κ* is calculated to be 0.4/min.

The reduction reaction occurs via relaying electrons from the donor BH_4_^-^ to the acceptor 4-NP after the adsorption of both onto the catalyst surface. The hydrogen atom, which is formed from the hydride, after electron transfer to the Au NPs attacks 4-NP molecules to reduce them. For comparison, the catalytic ability of the equal amount of γ-Fe_2_O_3_/mSiO_2_ is also studied. Without Au catalyst, the reduction reaction does not proceed, as evidenced by a nonvarying absorption spectrum.

To investigate the reusability of the γ-Fe_2_O_3_/Au/mSiO_2_ microspheres, we use a magnet to separate the catalysts from the solution and then rinse it with deionized water. Then, the microspheres are dispersed into deionized water for the next cycle of catalysis. As shown in Figure 6D, the γ-Fe_2_O_3_/Au/mSiO_2_ microspheres could be successfully recycled and reused for at least ten times within 10 min. The microstructures are well retained after the repeating catalytic processes.

## Conclusions

In summary, by employing a functionalized magnetic polymer microsphere template, we have successfully synthesized monodisperse, hierarchically mesoporous γ-Fe_2_O_3_/Au/mSiO_2_ microspheres with high surface area. Quaternary ammonium in the surface of the microspheres serves not only as a reducing agent but also as a protecting ligand, which makes the adsorption of gold nanoparticles simple and convenient. Gold nanoparticles are reduced *in situ* and incorporated into the matrix of porous microspheres. The resulting multicomponent microspheres have high magnetization and can be conveniently separated from the reaction solution using external magnetic fields. They exhibit excellent catalytic performance and high reusability for the reduction of 4-NP in the presence of NaBH_4_. This functional microsphere holds great promise as a novel gold-based catalyst system for various catalytic applications. Additionally, the approach for the fabrication of γ-Fe_2_O_3_/Au/SiO_2_ microspheres can be extended to synthesize other multicomponent nanostructures for advanced applications in chemical/biosensor, environmental detection, and electromagnetic devices.

## Abbreviations

4-AP: 4-aminophenol; 4-NP: 4-nitrophenol; Au NPs: gold nanoparticles; BET: Brunauer-Emmett-Teller; EDX: energy-dispersive X-ray spectroscopy; EGDMA: ethylene glycol dimethacrylate; GMA: glycidyl methacrylate; SEM: scanning electron microscope; TEM: transmission electron microscopy; TEOS: tetraethylorthosilane; TMA: trimethylamine; XRD: X-ray diffraction.

## Competing interests

The authors declare that they have no competing interests.

## Authors’ contributions

LR carried out the synthesis and characterization of porous silica microspheres. CT participated in the morphology characterization. LZ drafted the manuscript. JH and You Wang participated in the UV and TGA analyses. XZ participated in the XRD characterization. Yong Wang and BJ conceived of the study and helped draft the manuscript. MH and JZ participated in the design of the study. All authors read and approved the final manuscript.

## Supplementary Material

Additional file 1: Figure S1 (A-B) SEM images of commercially available porous P(GMA/EGDMA) microspheres. (C-D) TEM images of synthesized magnetic γ-Fe_2_O_3_ nanoparticles. Click here for file
