# Supplementary material for: Preparation of uniform magnetic recoverable catalyst microspheres with hierarchically mesoporous structure by using porous polymer microsphere template
Source: Nanoscale Res Lett. 2014 Apr 4;9(1):163. doi: 10.1186/1556-276X-9-163 (PMC4113952; doi:10.1186/1556-276X-9-163)
Supplement: Additional file 1: Figure S1 — (A-B) SEM images of commercially available porous P(GMA/EGDMA) microspheres. (C-D) TEM images of synthesized magnetic γ-Fe2O3 nanoparticles. [file 1556-276X-9-163-S1.doc]

Additional file 1

Support Information

Preparation of Uniform Magnetic Recoverable Catalyst Microspheres with Hierarchically Mesoporous Structure by Using Porous Polymer Microspheres Template

*Lianbing Ren,* ^†^ *Chao Teng,* ^†^ *Lili Zhu,* ^†^ *Jie He,* ^†^ *You Wang,^§^ Xinbing Zuo,^┸^ Mei Hong,* ^†,*^ *Yong Wang,* ^†,*^  *Biwang Jiang,* ^†,*^  *Jing Zhao*^†^, ^‡*^

*^†^*Shenzhen Key Lab of Nano-Micro Materials Research, School of Chemical Biology & Biotechnology, Peking University Shenzhen Graduate School, Shenzhen 518055, China.

*^§^*Shenzhen Middle School, Shenzhen, 518000, China.

*^┸^*Shenzhen State High-tech Industrial Innovation Center, Shenzhen, 518000, China.

*^‡^*State Key Laboratory of Pharmaceutical Biotechnology, School of Life Sciences, Nanjing University, Nanjing 210093, China.

Corresponding authors E-mail: ywang@pkusz.edu.cn; hongmei@pkusz.edu.cn; jiangbw@pkusz.edu.cn; jingzhao@nju.edu.cn;


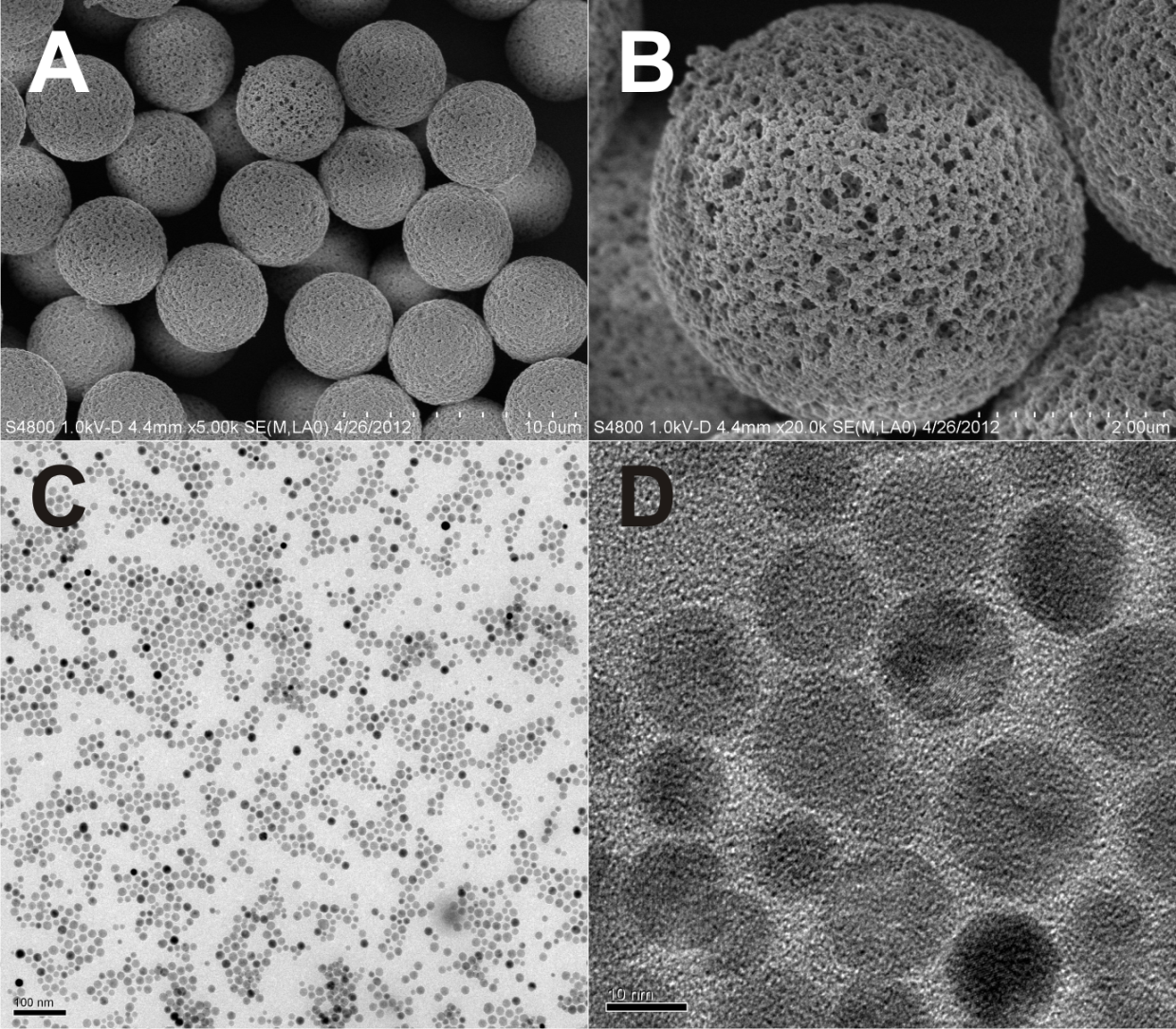


Figure S1 (A-B) SEM images of commercial available porous P(GMA/EGDMA) microspheres. (C-D) TEM images of synthesized magnetic γ-Fe_2_O_3_ nanoparticles.
